# Cell-Free Production of Soybean Leghemoglobins and Nonsymbiotic Hemoglobin

**DOI:** 10.1021/acssynbio.5c00197

**Published:** 2025-08-12

**Authors:** Amanda P. Rocha, Mariele A. Palmeiras, Marco Antônio deOliveira, Lilian H. Florentino, Thais R. Cataldi, Daniela M. de C. Bittencourt, Carlos A. Labate, Gracia M. S. Rosinha, Elibio L. Rech

**Affiliations:** † Embrapa Genetic Resources and Biotechnology/National Institute of Science and Technology - Synthetic Biology, Parque Estação Biologica, PqEB, Av. W5 Norte (final), Brasília, Distrito Federal 70770-917, Brazil; ‡ Max Feffer Laboratory of Plant Genetics—EMU, Department of Genetics, Luiz de Queiroz College of Agriculture, University of São Paulo, Avenue Pádua Dias 11, Piracicaba, São Paulo 13418-900, Brazil

**Keywords:** leghemoglobins, nonsymbiotic hemoglobin, cell-free system, protein production, hemoglobins, plant-based meat

## Abstract

Hemoglobins are heme proteins and are present in certain microorganisms, higher plants, and mammals. Two types of hemoglobin are found in legume nodules: leghemoglobin (LegH) or symbiotic and nonsymbiotic (nsHb). LegHs occur in high amounts in legume roots, and together with bacteroides, are responsible for the nitrogen fixation process. nsHb Class 1 proteins have very high affinity for O_2_ and are found in monocotyledons and legumes. LegH has attracted great interest in the vegetable meat industry owing to its organoleptic and nutritional properties. In this study, soybean LegHs A, C1, C2 and C3 and nsHb were produced via *Escherichia coli*-based cell-free systems (CFS) and their amino acid sequences were correctly synthesized. In addition, certain post-translational modifications were made, which were confirmed using liquid chromatography–mass spectrometry analysis. All LegHs produced in this system exhibited peroxidase activity and heme binding, which were correlated with their concentrations in the assays. Furthermore, all proteins were readily digested by pepsin within 1 min under analog digestion conditions. Thus, LegHs and nsHb proteins were produced in this study using cell-free systems, maintaining their functionality and digestibility. These findings suggest that they could serve as viable alternative food additives for plant-based meat.

## Graphical abstract

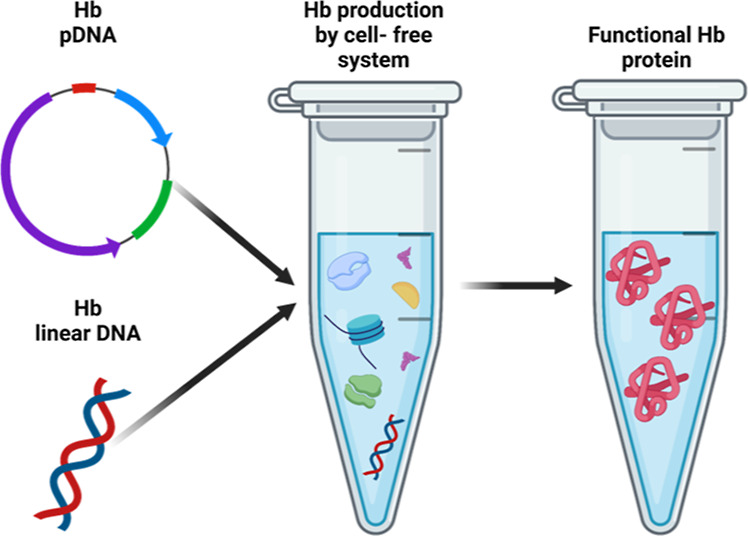

## 1 Introduction

Hemoglobins (Hbs) are heme proteins primarily responsible for the transport of O_2_. Three types of Hbs have been identified in plants: symbiotic Hbs, known as leghemoglobin (LegHs); nonsymbiotic Hbs (nsHbs); and truncated Hbs (tHbs).^1,2^ LegH is an essential protein for nitrogen fixation in the root nodules of legumes, promoting symbiosis between the host plant and bacteria of the genus *Rhizobium*.^1^ LegHs were the first globin proteins identified in plants and occur in high concentrations in the root nodules of soybean (*Glycine max*), which possesses five genes that are known to encode different LegH isoforms: LegH A, LegH pseudogene, LegH C1, LegH C2, and LegH C3.^1,2^

In contrast to LegHs, which are specific to legume nodules, nsHbs are found in a wide variety of plant species and tissues. These include soybeans in which nsHbs are expressed in various tissues such as the roots, leaves, and seeds, in addition to the symbiotic LegH found in nodules.^3^ While the exact roles of nsHbs are yet to be elucidated, studies suggest that they contribute to cellular energy production, especially under conditions of high energy demand or low O_2_ availability.^4^ Furthermore, nsHbs are expressed in the tissues of other plants, including the roots and seeds of rice, barley, and *Arabidopsis*, as well as in the leaves of alfalfa and the roots of cotton.^5^ This diverse expression pattern suggests that nsHbs play different roles depending on their location and the specific requirements of the plant.

Despite being discovered over 70 years ago,^6^ LegH has recently garnered significant attention for potential use as a food additive in plant-based meat products.^7^ This renewed interest stems from the growing demand for plant-based alternatives that closely mimic the sensory qualities of animal meat. Myoglobin, an iron-containing protein abundant in animal meat, plays a crucial role in the development of meat’s characteristic aroma, texture, and flavor during cooking. The heme group in myoglobin catalyzes certain reactions that transform amino acids, nucleotides, and sugars into complex flavor compounds. Hence, the plant-based meat industry is exploring the use of heme-containing plant proteins, such as LegH, to replicate these desirable qualities in their products.^7^

In LegH, polypeptide chains bind to a heme B cofactor, which is identical to the heme found in animal meat and has been a part of the human diet for centuries.^8,9^ Importantly, the bioavailability of the iron in LegH is the same as that in bovine Hb,^10^ which is crucial as heme iron constitutes approximately 95% of the body’s iron store and is the primary source of iron for 67% of people in developed countries.^11^ Therefore, LegH is a potentially valuable source of dietary iron and offers several advantages as a possible food additive. Its amino acid sequence is not homologous to any known human allergens or toxins; moreover, it is easily digested by pepsin under simulated gastric conditions.^12^

Impossible Foods produces soybean LegH C2 using the yeast *Pichia pastoris* as the host organism. This LegH serves as a key ingredient in their plant-based meat products, contributing to the characteristic flavor and aroma.^13^ The United States Food and Drug Administration has authorized the use of this LegH as a color additive in plant-based burgers, with a maximum permitted limit of 0.8%.^14,15^ The commercially produced soybean LegH C2, known as “LegH Prep”, exhibits a purity of approximately 65%. The remaining 35% comprises other proteins derived from the *P. pastoris* host.^16^

LegH has also been produced in *Escherichia coli*,^17,18^ *Kluyveromyces marxianus*,^19^ and *Corynebacterium glutamicum* *.* ^20^ However, only a few nsHbs have been expressed heterologously, e.g., type 1 and type 2 nsHbs from rice. A previous study aimed to elucidate the biological functions of nsHbs, which are thought to play a significant role in the expression and utilization of O_2_ in plants. The in vivo activities of rice nsHb-1 and nsHb-2 were investigated by analyzing their effects on the growth of *E. coli* TB1. The findings revealed that growth inhibition was more pronounced when nsHb-2 was synthesized compared with nsHb-1, suggesting that these Hbs have distinct in vivo roles in rice.^21^

Synthetic biology enables the optimization and simplification of biological processes, for example, by producing proteins in simple systems that mimic complex cells, utilizing only the essential machinery required for protein synthesis. This approach allows substrates and energy to be directed only toward target protein production, while permitting the possible production of potentially toxic proteins, such as LegH. Studies in *E. coli* have shown that the iron-containing heme groups in LegH can promote the formation of free radicals within the bacterial cells.^17^ This oxidative stress can damage cellular components and potentially hinder LegH production. Therefore, enabling its production in synthetic biological systems would be a promising tactic.

Cell-free systems (CFSs) are rapidly emerging as a versatile platform for protein biosynthesis, offering a promising alternative to conventional cell-based methods. Unlike protein expression in living cells, which can be limited by cellular growth and complex regulatory networks, CFSs operate in a simplified environment.^22^ This method eliminates the constraints imposed by cell membranes and complex cellular processes, permitting the precise control of reaction conditions and the optimization of protein production.^22^ Moreover, these systems facilitate the production of proteins that may be toxic or difficult to express in living cells, such as membrane proteins and those involving complex post-translational modifications (PTMs).^23^

In this system, proteins are produced rapidly and efficiently using crude cellular extracts derived from prokaryotic or eukaryotic cells that contain the required native cellular transcriptional and translational machinery.^24^ The major components of the cell-free protein synthesis reaction mixture are as follows: template DNA encoding the target protein (circular or linear); a crude cellular extract; substrates for transcription and translation, including nucleotides and amino acids; and the components needed for ATP regeneration.^25^ Extracts prepared from different organisms vary in terms of expression yield and the difficulty of synthesizing more complex proteins. For example, eukaryotes, which make post-translational changes, do not yield high levels of protein. Therefore, expression systems that utilize prokaryotic organisms provide a higher yield. However, these systems may not be suitable for producing certain mammalian proteins, especially those that require PTM for proper function.^26^

Protein expression using CFSs has become increasingly attractive compared with conventional methods owing to the ease with which proteins can be purified after expression. The reason is that such systems lack a cell wall, rendering the lysis step unnecessary. The process is thus much more agile and practical.^23^ Moreover, energy and metabolic expenditure can be directed only toward producing the protein of interest as energy expenditure toward the cell’s functioning and survival is not required.^27^ Finally, the cell-free expression method can produce proteins that are lethal to the cellular environment as they would inhibit transcription and translation, making the process of protein synthesis unfeasible.^26^

The increasing accessibility of CFSs could largely be attributed to the availability of both commercial and homemade kit resources. Commercial kits produced by various biotechnology companies include cell extracts, enzymes, amino acids, and energy sources, along with optimized protocols and DNA templates,^28^ providing a standardized approach. On the contrary, the main advantage of homemade kits is the dramatic reduction in cost (up to 30×^29^), while allowing greater flexibility and customization.^27^

In this work, we demonstrated for the first time that soybean LegHs (LegH A, C1, C2, and C3) and an nsHb can be effectively produced using an *E. coli*-based CFS. Moreover, these synthetically produced proteins exhibited peroxidase activity, possessed a heme group, and were completely digested when exposed to simulated gastric fluid, thus increasing the number of heme proteins with potential use as additives in plant-based meat products.

## 2 Results and Discussion

### 2.1 Cell-Free Synthesis of LegHs and nsHb Proteins

Four types of LegHs, namely, LegH A, LegH C1, LegH C2, and LegH C3 and one nsHb, namely, nsHb, are believed to be derived from soybean nodules.^30^ However, a recent study has identified five LegHs and two nsHb in soy.^2^ Wiborg et al. analyzed the sequences and found that four of the LegH proteins possess several conserved regions, which explains the similarity among them.^31^ The difference between the sequences of LegH A and LegH C3, the most distinct LegHs, is only 8%,^31^ whereas approximately six amino acids differ in the sequences of the four LegHs analyzed (Figure S1). nsHb is found in soybean nodules and other parts of the plant, such as embryos, leaves, and roots,^3,32^ and has a more distinct amino acid sequence than LegHs (Figure S1).

CFS is a method that utilizes only the essential components necessary for transcribing and translating DNA into protein in vitro, operating within a simple, open, and controlled system. This approach creates a versatile and easily manipulable system compared with living cells, enabling the addition of desired components while eliminating unwanted byproducts that could inhibit protein synthesis. Furthermore, the transcription and translation capabilities of the CFS can accommodate various formats, including batch, continuous flow, and continuous exchange, thereby augmenting protein synthesis and increasing the yield.^29^ Notably, CFS allows the production of cytotoxic and transmembrane proteins in a controlled and optimized environment, which can be challenging to achieve in living cells.^33^ Of the various options, the *E. coli* machinery has shown the highest efficiency in target protein synthesis.^29^

The DNA sequences of four LegHs (A, C1, C2, and C3) and one nsHb from soybeans were synthesized into a pET28a vector containing the promoter for T7 polymerase, RBS sequence, and 6× Histidine tag at the C-termini of each Hb (Figure S1). From these components, all desired proteins were successfully produced using an *E. coli*-based CFS prepared in our laboratory, which operated for 16 h at 28 °C. Bands corresponding to LegHs (approximately 16 kDa with His-Tag tail) and nsHb (18 kDa with His-Tag tail) were visualized using sodium dodecyl sulfate-polyacrylamide gel electrophoresis (SDS-PAGE) of raw CFS reaction extracts (data not shown). Electrophoresis of His-Tag–purified proteins revealed two bands, one corresponding to the LegH (approximately 16–20 kDa) and one corresponding to the His-tagged T7 RNA polymerase included in the CFS reactions (approximately 100 kDa) (data not shown). The successful production of LegHs and nsHB using CFS and purification by affinity chromatography was also confirmed with immunoblotting analysis using antibodies against the 6xHis-tag (Figure 1A). For the purified fractions, specific bands corresponding to the LegHs were identified, asserting the efficiency of purification (Figures 1A,B). An average of 0.5 mg/mL was obtained for both LegHs and nsHb in our optimized CFS.

**Figure 1:**
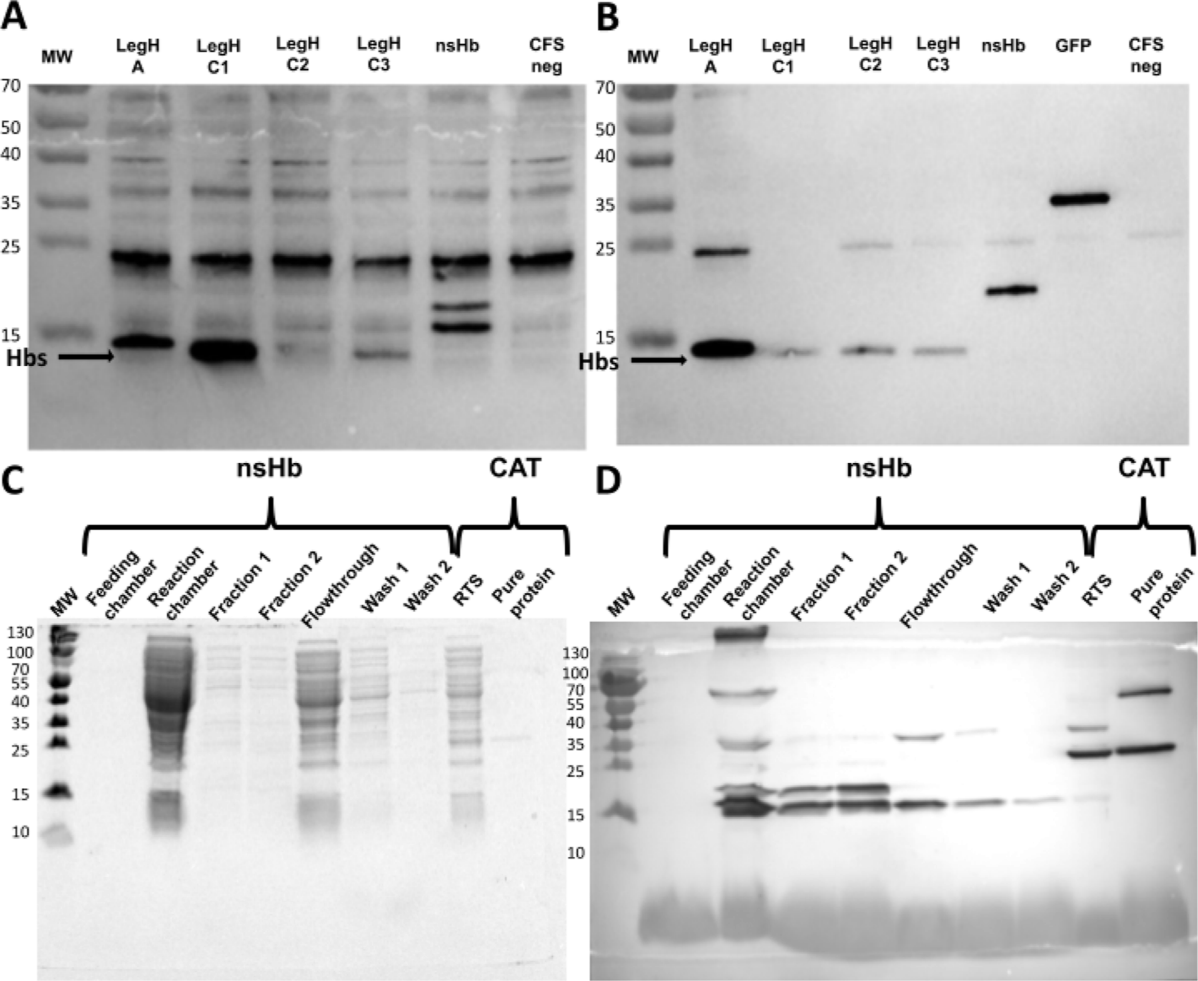
Production of LegH A, C1, C2, and C3 and nsHb proteins in a CFS. (A) Western blot analysis of cell-free expressed crude extracts of hemoglobins (LegHs, ≈15 kDa; nsHB, ≈18 kDa). (B) Western blot analysis of cell-free expressed purified hemoglobins. (C) Western blot analysis of nsHB protein produced using a medium-scale commercial continuous-exchange cell-free (CECF). (D) A Coomassie stained SDS-PAGE analysis of purified nsHB produced by CECF.

As nsHb has often produced the most intense band in Western blot analyses, we wondered whether it can also be made using medium-scale CFS. The commercial kit RTS 500 ProteoMaster from Rabbit Biotechnology (no. BR1400201), which is based on a continuous-exchange cell-free (CECF) protein synthesis system, was used for this purpose. This system comprises an inner chamber for protein production (reaction chamber), which is fed by an external chamber with an excess of substrates (feeding chamber). This setup also allows byproducts to pass through a semipermeable membrane, preventing saturation of the reaction chamber. The CECF device used is represented in Figure S2.

nsHb was successfully produced in medium-scale CECF for the first time, generating approximately 0.5 mg of the target protein in a 1 mL reaction with an *E. coli* extract. However, purification was unsatisfactory because several bands were observed in SDS-PAGE with Coomassie blue staining and in immunoblotting analysis, which also revealed the loss of proteins during the wash steps (Figure 1C,D). Therefore, chromatographic conditions must be optimized during the purification of nsHB in medium-scale systems to enhance production.

### 2.2 Liquid Chromatography–Mass Spectrometry (LC–MS) Analysis of Hbs Produced in the CFS

Mass spectrometric analysis of the LegHs A, C1, C2, and C3 and nsHb samples found 13, 20, 10, 14, and 7 peptides following trypsin digestion, covering 93%, 99%, 73%, 77%, and 40% of the amino acid sequence, respectively, with a false discovery rate (FDR) of 0.0% (Figure 2). Approximately 9, 9, 4, 2, and 6 unique peptides were identified using LC–MS analysis in the LegH A, C1, C2, and C3 and nsHb samples, respectively. The findings indicated that CFS-produced LegHs and nsHb exhibited the correct amino acid sequence. The PTM N-terminal acetylation, reported in another study,^38^ could not be identified in our synthesized LegHs, possibly because of peptide digestion before analysis. Detailed data for each identified peptide for coverage of each Hb are shown in Table S1. Analysis of the LC–MS results revealed the most common contaminating proteins present in our purified samples. The most common contaminating protein was the large ribosomal subunit protein uL3 (RL3), followed by the chaperone protein DnaK and T7 RNA polymerase (RPOL). Table 1 lists the nine most abundant proteins found in all or at least 3–4 experimental samples.

**Figure 2:**
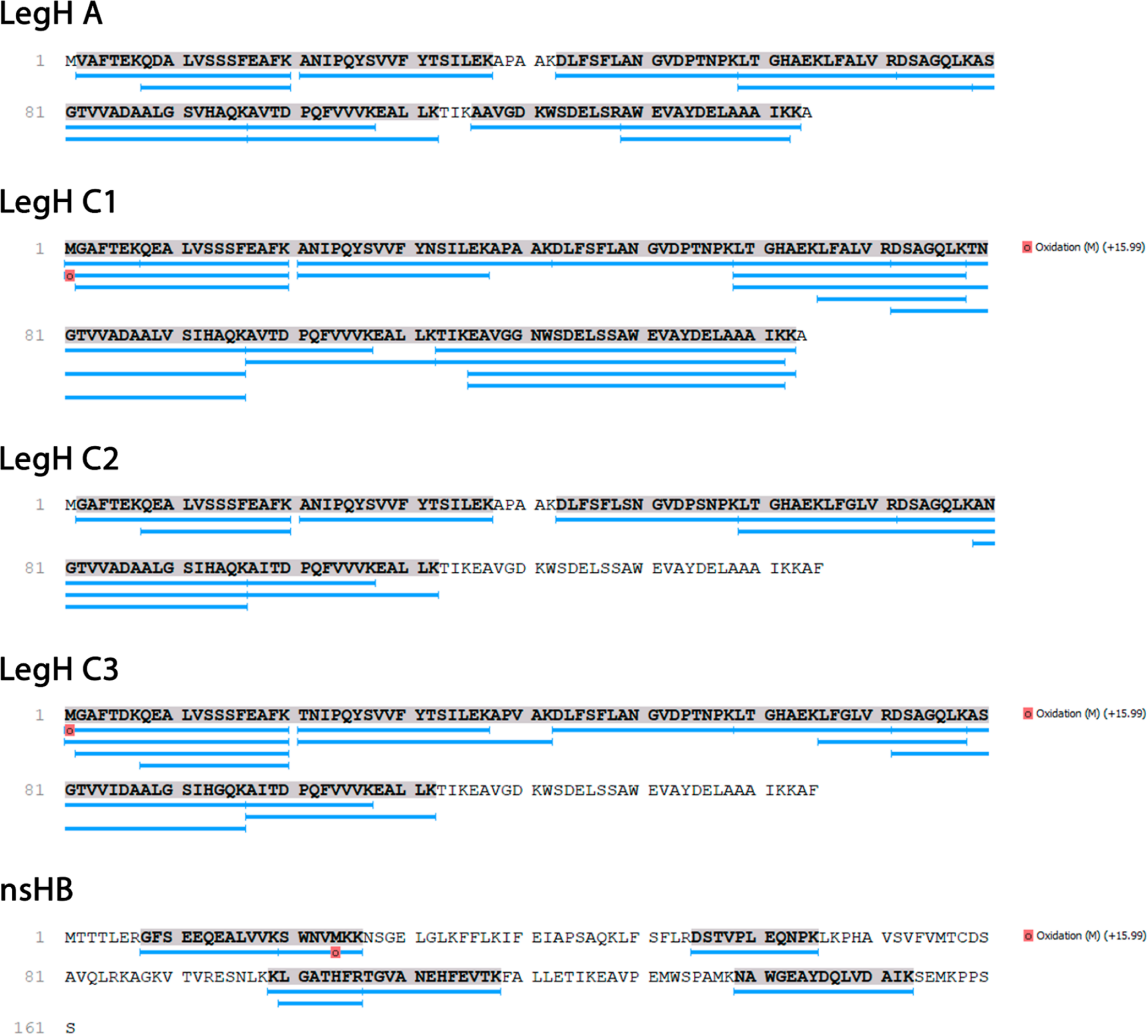
LegH A, C1, C2, and C3 and nsHb were produced in a CFS with their correct amino acid sequences. The digested proteins were subjected to LC–MS and analyzed using the PEAKS BD software. The peptides detected from each sample were filtered using an FDR of up to 1% and aligned with the LegH A, C1, C2, and C3 and nsHb amino acid sequences provided by the UniProt database. The blue lines below each amino acid sequence indicate trypsin-generated peptides identified using LC–MS; bold and highlighted letters represent the peptide coverage of original sequences. Amino acids for which PTMs, such as oxidation, have been identified are shown as red squares under the respective amino acid.

**Table 1:** Most Abundant Proteins from *E. coli* in Purified Hbs Produced by the Cell-Free System Found in LC–MS

protein ID^a^	accession^b^	–10 lg *P* ^c^	coverage (%)^d^	peptides number^e^	unique peptides^f^	description
1061	sp|B7L4K9|RL3_ECO55	463.42	72	19	19	large ribosomal subunit protein uL3
55	sp|A7ZHA4|DNAK_ECO24	583.99	71	52	51	chaperone protein DnaK
21	sp|P00573|RPOL_BPT7	704.22	75	82	82	T7 RNA polymerase
4	sp|P00490|PHSM_ECOLI	635.42	81	70	70	maltodextrin phosphorylase
52	sp|Q8XEG2|GLMS_ECO57	665.46	72	44	44	glutamine--fructose-6-phosphate aminotransferase
5	sp|P0A6N2|EFTU_ECOL6	563.88	81	31	31	elongation factor Tu
1014	sp|A7ZUD3|TPIS_ECO24	474.13	78	21	21	triosephosphate isomerase
852	sp|B1IQH2|RS2_ECOLC	496.26	85	25	25	
96	sp|P35340|AHPF_ECOLI	511.53	60	29	29	alkyl hydroperoxide reductase subunit F

a Protein ID from the UniProt database.

b Unique identifier assigned to each protein sequence entry in the UniProt database.

c Protein reconstruction score by Peaks software, used in LC/MS analysis.

d Percentage of a protein’s amino acid sequence identified by the peptides detected by LC/MS.

e Number of detected peptides identified by spectrophotometer and used to identify the protein.

f Number of unique peptides identified from the specific protein.

### 2.3 LegHs and nsHb Exhibit Peroxidase Activity

LegHs are known to display pseudoperoxidase activity, which may play a role in protecting against oxidant radicals in nodules, forming compounds with hydrogen peroxide and reducing organic peroxides.^19,39^ In this study, 40 μg/mL of the synthesized LegH A, C1, C2, and C3 and nsHb demonstrated dose-dependent peroxidase activities of approximately 7, 5, 2, 4, and 1 U/mL, respectively, with LegH A showing the highest activity. Therefore, 20–40 μg/mL of LegH and nsHb produced in CFS are sufficient to observe peroxidase activity. Nonetheless, peroxidase activity could not be detected in concentrations of 5–10 μg/mL of Hbs, suggesting an activity detection limit at approximately these concentrations (Figures 3 and S3).

**Figure 3:**
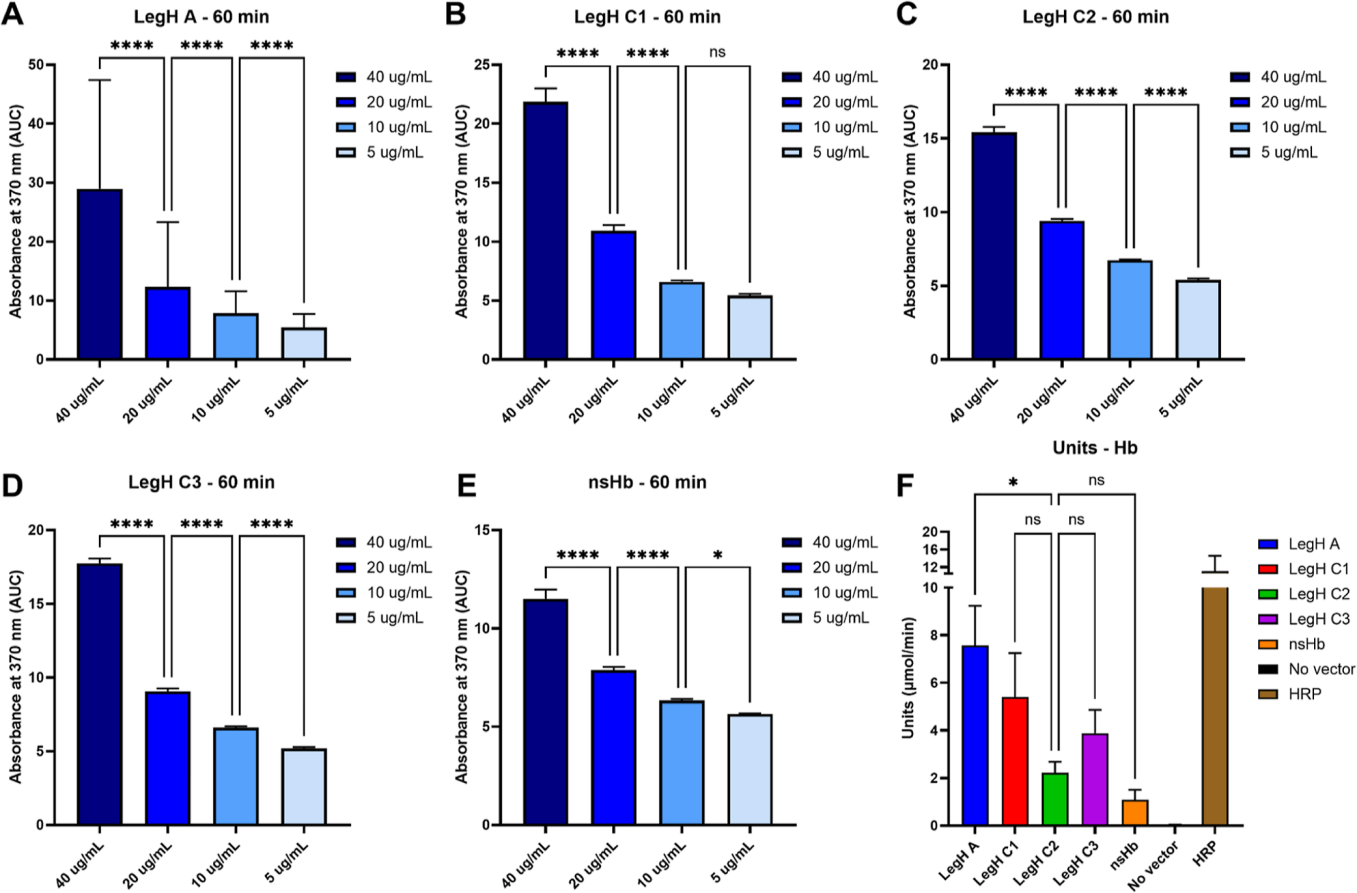
LegH A, C1, C2, and C3 and nsHb produced from the CFS exhibited peroxidase activity. The area under the curve was determined based on the action of 40, 20, 10, and 5 μg/mL of LegH A (A), C1 (B), C2 (C), and C3 (D) and nsHb (E). Units of enzymatic activity with 40 μg/mL of LegH A, C1, C2, and C3 and nsHb (F). Values shown are means and standard error from four independent experiments performed in duplicate. *P*-values were calculated using one-way ANOVA, with multiple comparisons corrected using Tukey’s test. Significance was represented by *­(*p* < 0.05); **­(*p* < 0.01); ***­(*p* < 0.001); ****­(*p* < 0.0001). NS: nonsignificant.

Soy LegH C2 produced in *P. pastoris* has been reported to exhibit peroxidase activity between 280 and 430 U/mg.^40^ Shao et al. attested that LegH C2 secreted by engineered *P. pastoris* presented a peroxidase activity of approximately 400 U/mg at 250 mg/L, corroborating our results.^41^ LegH produced in *E. coli* has also been documented to exhibit peroxidase activity.^17^ Furthermore, comparison of the LegHs produced using the CFS in our study revealed that the peroxidase activity of LegH A was almost 4-fold higher than that of LegH C2, which is being used by the food industry to produce plant-based meat.

Other proteins have also been successfully produced using CFSs and are bioactive, such as insulin,^34^ invasion plasmid antigens B (IpaB),^35^ serratiopeptidase,^36^ and secretory leukocyte protease inhibitor (SLPI),^37^ implying that this method enables the production of native proteins when the appropriate organism is used as the extract source to provide certain PTMs.

### 2.4 LegHs and nsHB Produced Using CFS Contain a Heme Group

The heme group, similar to that found in myoglobin, may become denatured and exposed when subjected to high temperatures during cooking. This denaturation promotes reactions that generate complex compounds responsible for the aroma, flavor, and texture of cooked meat. The presence of the heme group in soybeans and other legumes has sparked considerable interest in the plant-based meat industry, driving efforts to create meat analogs that cater to vegetarian and vegan consumers.^7^

The LegHs and nsHb produced using CFS were confirmed to possess a heme group. LegH A, C1, C2, and C3 and nsHb exhibited a similar heme concentration in a dose-dependent manner compared with Hb concentration, with an average of 2 μM of heme with 30 μg/mL of protein (Figure 4A), corresponding to approximately 53% of the heme-binding ratio, as suggested by Shao et al.^41^ Although some heme content were not significant different in distinct Hbs concentration, correlation coefficients using Pearson’s test between heme content and LegH at different protein concentrations (40, 30, 10, and 6 μg/mL) demonstrated that LegH A, C1, and C3 and nsHb exhibited significant *P*-values (0.0031, 0.0410, 0.0014 and 0.017, respectively) and R squared nearly to 1 (0.9938; 0.9197; 0.9971 and 0.9768, respectively), demonstrating that as Hb concentration increased also heme content increased. On the other hand, no vector CFS reactions did not present correlation between protein concentration and heme content (*R* ^2^ = 0.003974 and *P* value = 0.9598). These data suggest that LegHs A, C1, C3 and nsHB produced in the CFS system contain heme group with a better correlation to legH C2, already used as a food additive in plant-based meat.

**Figure 4:**
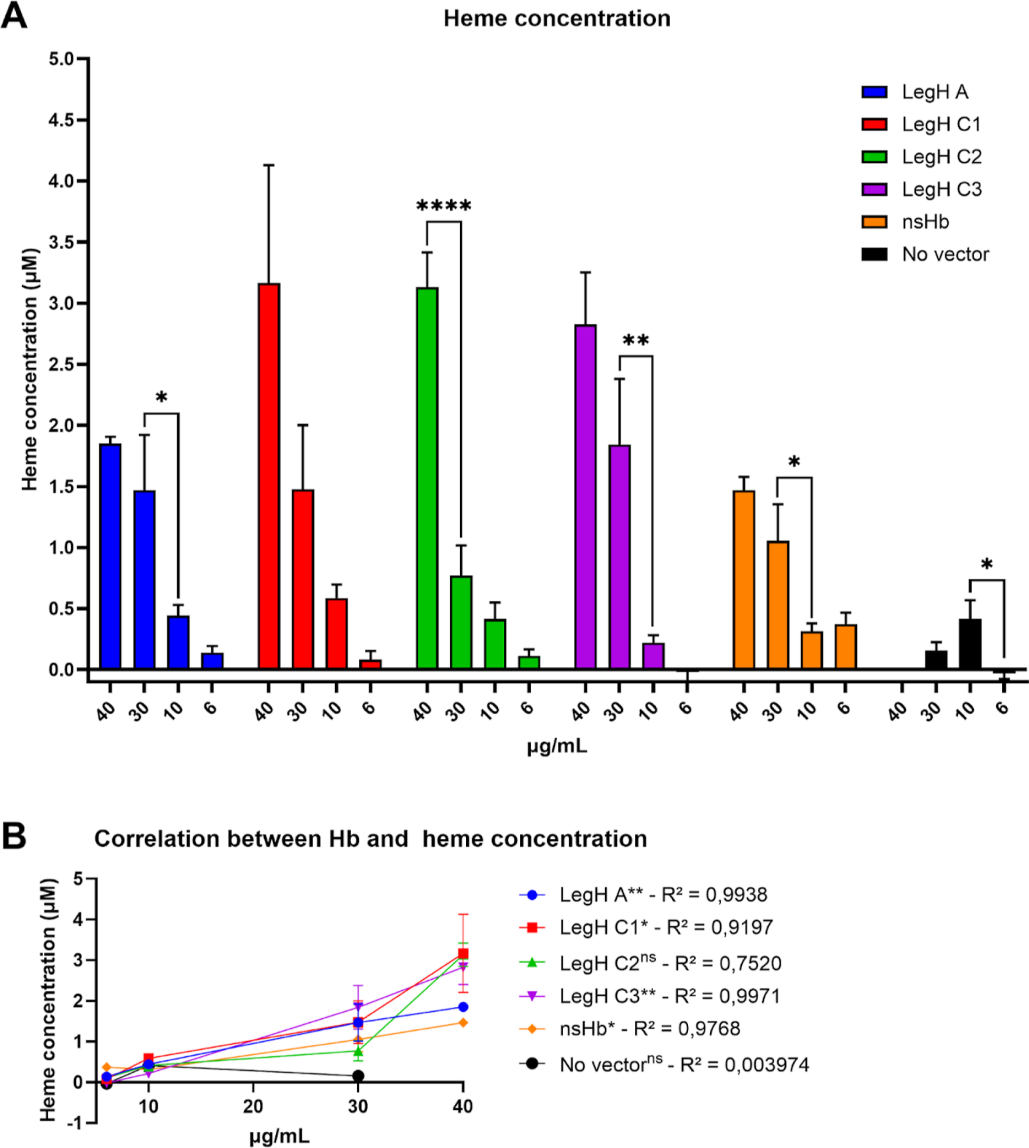
LegH A, C1, C2, and C3 and nsHb produced from the CFS have a heme group in their structure. (A) The heme concentration was measured from the absorbance and represented as the mean and standard error from three independent experiments performed in duplicate. *P*-values were calculated using one-way ANOVA, with multiple comparisons corrected using Tukey’s test. Significance was represented by *­(*p* < 0.05); **­(*p* < 0.01); ***­(*p* < 0.001); ****­(*p* < 0.0001). NS: nonsignificant. (B) Correlation graphic from heme concentration and Hb protein concentration, showing *R* ^2^ values for each data set. Only significant comparisons are shown in the graphs; other comparisons were nonsignificant. *P*-values were calculated using the Pearson linear correlation coefficient. Significance was represented by *­(*p* < 0.05); **­(*p* < 0.01); ***­(*p* < 0.001); ****­(*p* < 0.0001). NS: nonsignificant.

This work also verified the presence of heme groups in LegHs other than LegH C2 and nsHB. Only LegHs purified directly from soybean root nodules^10^ and LegH C2 produced in *P. pastoris* ^41,42^ have so far been analyzed for the presence of the heme group. It is worth mentioning that the heat process did not interfere with the heme iron of LegHs,^10^ suggesting that cooking is not likely to affect these proteins when used as food additives. Adjusting the CFS and allowing hemin supplementation may increase the heme concentration in the synthesized protein, as reported by other studies that have observed this increment in heme proteins.^43,44^

### 2.5 In Vitro Pepsin Digestibility Test

Protein digestion and degradation by pepsin have been recommended for in vitro testing of allergenicity potential, with the aim of extrapolating to human tolerance.^45–49^ To analyze the LegHs and nsHB produced in our CFS, these proteins were incubated with pepsin in a simulated gastric fluid of pH 2.0, mimicking mammalian stomach conditions. All Hb bands disappeared from the Western blots after 1–50 min of digestion with pepsin, suggesting their digestion under mammalian stomach conditions (Figure 5). This finding corroborated previous results showing that a LegH C2 preparation (mixture containing the soy LegH C2 isoform, residual *Pichia* proteins, and added food-grade stabilizers)^42^ and a purified LegH C2 ^50^ were digested within 2 min by pepsin.

**Figure 5:**
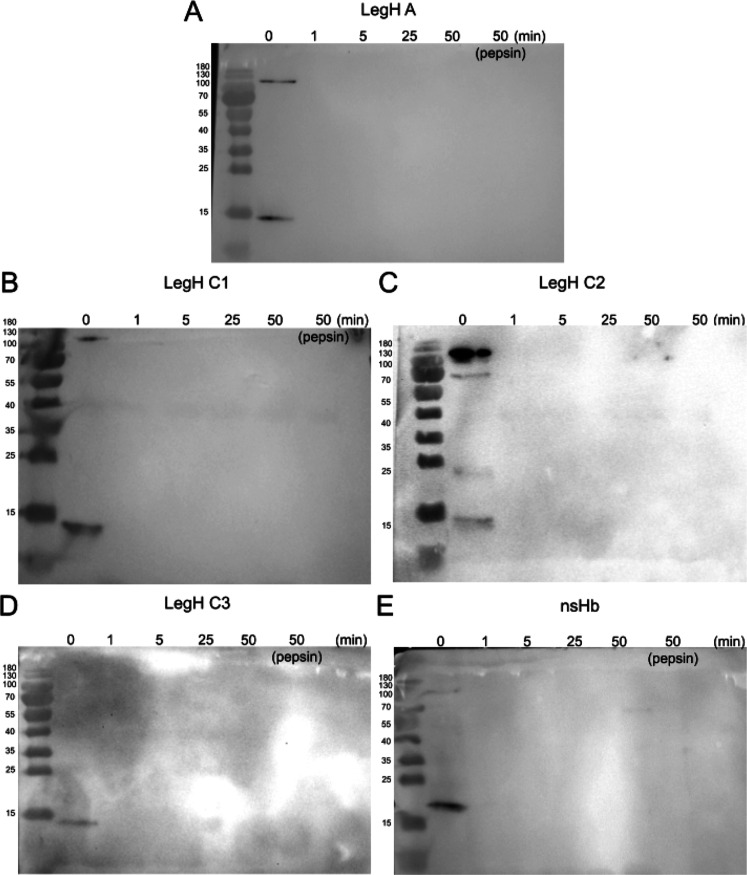
LegH A, C1, C2, and C3 and nsHb produced in CFS are digested using pepsin. Hbs were incubated with pepsin (10 U/μg of LegH) in a simulated gastric fluid (SGL) of pH 2.0 at 37 °C for 0, 1, 5, 25, and 50 min. Digestion products were separated in SDS–PAGE and submitted to Western blot analysis (A–E).

## 3 Conclusion

Soybean LegH variants (C1, C2, C3, and A) and nsHb can be efficiently produced using both commercial and laboratory-prepared CFSs, thereby expanding the synthetic biology toolkit for generating these proteins essential for plant-based meat cultivation. These Hbs were successfully synthesized on small and medium scales, with the latter achieved via the CECF system. Proteomic analysis confirmed that LegHs and nsHb were produced with the correct amino acid sequences. Furthermore, LegH variants and nsHb synthesized using the CFS demonstrated pseudoperoxidase activity and heme-binding ability, emphasizing their role in iron storage. All examined soybean Hbs were rapidly digested by pepsin within 1 min, suggesting their potential as safe food additives for plant-based meats while minimizing the risk of intolerance. Therefore, all tested LegHs and nsHb produced using the CFS retained the essential properties for their application as plant-based meat additives.

## 4 Methods

### 4.1 Vector Design and Synthesis

The gene sequences of LegHs and nsHb were obtained from NCBI (LegH A Gene ID: 100527427, LegH C1 Gene ID: 100785236, LegH C2 Gene ID: 100527379, LegH C3 Gene ID: 100527391, and nsHb Gene ID: 102661758), codon-optimized for *E. coli*, and cloned into pET28a by EPOCH Life Science Inc. (Missouri City, TX, EUA). Specifically, the genes were inserted between the NcoI and XhoI restriction sites in the multiple cloning site of the plasmid pET28a­(+). To facilitate protein capture and purification, LegHs and nsHb were expressed with a C-terminal His6-Tag.

Subsequently, the recombinant plasmids were transformed into the DH5α *E. coli* strain, and a single colony was selected for expansion culture. The plasmid DNA encoding LegH A, LegH C1, LegH C2, LegH C3, and nsHb was extracted using the plasmid Maxi kit (Qiagen). Sequences and maps for all plasmids are presented in Supporting Information_Soybean_1.

### 4.2 Cell-Free Extract Preparation

Cell-free extract and reagents used were prepared as previously described^50^ with necessary modifications. Briefly, to obtain a cell-free extract, *E. coli* Rosetta 2 cells were initially cultured in 50 mL of 2× YPG medium supplemented with chloramphenicol at 37 °C and 180 rpm for 16 h. A 5 mL aliquot of this culture was then transferred to 750 mL of 2× YPG medium in a 2 L Erlenmeyer flask and incubated at 30 °C and 180 rpm until the optical density at 600 nm reached 0.5. The cells were harvested, washed, and lysed via sonication on ice (15% amplitude; 10 s ON and 15 s OFF) with a total energy input of 2.7 kJ. The lysate was centrifuged at 15,000 g for 30 min at 4 °C. The supernatant containing the cell-free extract was collected and immediately frozen for storage.

The amino acid mix was prepared using a 20 mM stock solution from each of the 20 standard amino acids: alanine, arginine, asparagine, aspartic acid, cysteine, glutamic acid, glutamine, glycine, histidine, isoleucine, leucine, lysine, methionine, phenylalanine, proline, serine, threonine, tryptophan, tyrosine, and valine. Each amino acid was dissolved in a 400 mM KOH solution whose pH was adjusted to 6.6.

An energy mix of 10× concentration was prepared for the cell-free protein synthesis reaction. This solution contained 500 mM HEPES (pH 8.0), 15 mM ATP, 15 mM GTP, 9 mM CTP, 9 mM UTP, 0.68 mM folinic acid, 2 mg/mL *E. coli* tRNA mixture, 3.3 mM NAD, 2.6 mM coenzyme A, 15 mM spermidine, 40 mM sodium oxalate, 7.5 mM cAMP, and 300 mM 3-phosphoglyceric acid.

To acquire T7 polymerase DNA, the *E. coli* strain BL21, previously transformed with the vector UMN 1396p (Pt7-911Q, generously provided by Kate Adamala, University of Minnesota), was cultured in 20 mL of LB medium containing ampicillin at 37 °C and 180 rpm for 16 h. Subsequently, 5 mL of these cells were inoculated into 500 mL of LB medium containing ampicillin and incubated at 37 °C and 250 rpm until the optical density reached 0.5 at 600 nm. At this point, 1 mM IPTG was added to the culture. After 3 h of incubation, the culture was cooled and centrifuged for 10 min at 3700 rpm and 4 °C. Later, the pellet was lysed with lysis buffer (50 mM HEPES KOH pH 7.6, 1 M NH_4_Cl, and 10 mM MgCl_2_), sonicated four times (15% amplitude [approximately 7 W]; cycles of 15 s ON/15 s OFF until 2 kJ was reached), and again centrifuged for 45 min and 15,000 g at 4 °C. The supernatant was recovered and purified using a Ni-NTA agarose slurry in a chromatographic column. The purified T7 polymerase protein was dialyzed using Slyde-A-lyzer MWCO 30 kDa cassettes and stored at −20 °C in 50% glycerol until use. The purification of T7 polymerase was verified using SDS-PAGE and Western blotting, with a His-Tag antibody confirming a protein of 106 kDa.

### 4.3 Cell-Free Protein Synthesis of LegHs and nsHb

LegHs and nsHb were produced in vitro using a reaction mixture comprising 12 mM M-glutamate, 140 mM K-glutamate, 1 mM DTT, energy mix diluted 1:10, 2 mM of each amino acid, 1 U/μL murine RNA inhibitor, 1.5 μM T7 RNApol, and cell-free extract from *E. coli* Rosetta 2 diluted 1:3. The pDNA concentration ranged from 5 mM to 50 mM. The standard reaction conditions were 50–100 μL with 16 h of incubation at 28 °C, and the samples were maintained at 4 °C for short-term storage or at −20 °C for long-term storage until used for downstream applications. GFP pDNA was used as the positive control.

To produce nsHb on a medium scale, CFSs from RTS 500 ProteoMaster *E. coli* HY (BiotechRabbit), which utilizes a CECF system, were used according to the manufacturer’s instructions. Reaction mixtures of 1 mL were incubated at 32 °C and 800 rpm for 20 h. pDNA weighing 20 μg was used per reaction. The chloramphenicol acetyltransferase vector was utilized as the positive control.

For the purification of LegHs and nsHB in a small scale, His-Spin miniprep from Zymo Research (Cat no. P2002) was used according to the manufacturer’s guidelines. For the purification of LegH on a medium scale, HisPur Ni-NTA resin (Cat. no. 88221) was used according to the manufacturer’s protocol. Briefly, incubated CFS reaction mixtures were mixed with lysis buffer containing 10 mM imidazole, added to spin columns containing Ni-NTA resin, and washed twice with the same buffer. Subsequently, His-tagged proteins were eluted by adding 300–500 μL of elution buffer containing 300 mM imidazole. Purified proteins were dialyzed and concentrated using an Amicon Ultracel 3K membrane. The proteins were centrifuged thrice at 15,000 rpm for 20 min using 500 μL of PBS as the exchange buffer.

### 4.4 Western Blot Analysis

To confirm the production of LegHs and nsHb, both whole CFS reaction mixtures and purified proteins were denatured in mPAGE 4× LDS buffer containing 25 mM β-mercaptoethanol at 70 °C for 5 min and subjected to SDS-PAGE in a BIS-Tris gel with 15% polyacrylamide. Electrophoresis was performed for approximately 2 h in a MOPS buffer. Then, proteins from the gel were transferred to a PVDF membrane using a semidry system for 30–40 min, applying 20 V. Subsequently, the membranes were blocked with 5% BSA for 30 min at room temperature, incubated with anti-6×-His conjugated with either alkaline phosphatase (Cat no. 46-0284) or HRP (Cat no. A7058) at 4 °C for 16 h. The proteins were visualized using a 1:400 BCIP/NBT solution (Cat no. 72091) in Tris–HCl buffer pH 9.2 for 20–60 min or with Immobilon Forte Western HRP substrate (Cat no. WBLUF0500) and examined using chemiluminescence in the iBright Image System (Invitrogen).

### 4.5 LC–MS and Search Parameters in Public Databases

Purified and desalted LegHs and nsHb were resuspended to a final concentration of 2 μg/mL in 50 mM ammonium bicarbonate with Amicon Ultra 0.5 mL. The proteins were denatured by adding 0.025–0.1% RapiGest SF, vortexed, and incubated at 80 °C for 15 min before adding 5 mM DTT and heated at 60 °C for 30 min. Subsequently, 15 mM iodoacetamide was added, followed by a second 30 min incubation. Then, 1 μg/μL of trypsin was added, and the mixture was further incubated at 37 °C for 16 h. Trifluoride acid was added to hydrolyze RapiGest SF, followed by centrifugation at 18,000 g and 6 °C, and the supernatant was recovered, concentrated, and purified in a Reversed-Phase ZipTip C18, P10 (Cat no. ZTC18M096, Millipore). The samples were resuspended in 0.1% formic acid and analyzed using a hybrid trapped ion mobility spectrometer–quadrupole time-of-flight mass spectrometer (timsTOF Pro, Bruker Daltonics), assisted by a nano Elute nanoflow chromatographic system (Bruker Daltonics) and an ion source (CaptiveSpray). LC–MS was performed on a NanoElute (Bruker Daltonik) system coupled online to a hybrid TIMS-quadrupole TOF mass spectrometer^51,52^ (timsTOF Pro, Bruker Daltonik, Germany) via a nanoelectrospray ion source (Captive Spray, Bruker Daltonik). For long gradient runs (2 h total run), approximately 200 ng of peptides were separated on an Aurora column 25 cm × 75 μm ID, 1.9 μm reversed-phase column (Ion Opticks) at a flow rate of 300 nL min^–1^ in an oven compartment heated to 50 °C. To analyze samples from whole-proteome digests, a gradient starting with a linear increase from 2% B to 17% B over 60 min was used, followed by further linear increases to 25% B in 30 min and to 37% B in 10 min, and finally, to 95% B in 10 min, which was held constant for 10 min. The column was equilibrated using four volumes of solvent A. The mass spectrometer was operated in the data-dependent PASEF^53^ mode with 1 survey TIMS-MS and 10 PASEF MS/MS scans per acquisition cycle. An ion mobility range from 1/*K*_0_ = 1.6 to 0.6 Vs cm^–2^ was analyzed using equal ion accumulation and ramp times of 100 ms each in the dual TIMS analyzer. Suitable precursor ions for MS/MS analysis were isolated in a window of 2 Th for *m*/*z* < 700 and 3 Th for *m*/*z* > 700 by rapidly switching the quadrupole position in sync with the elution of precursors from the TIMS device. The collision energy was lowered stepwise as a function of increasing ion mobility, starting from 20 eV for 1/*K*_0_ = 0.6 Vs cm^–2^ and 59 eV for 1/*K*_0_ = 1.6 Vs cm^–2^. The *m*/*z* and ion mobility information were utilized to exclude singly charged precursor ions with a polygon filter mask. Furthermore, “dynamic exclusion” was used to avoid resequencing of precursors that reached a “target value” of 20,000 au The ion mobility dimension was calibrated linearly using three ions from the Agilent ESI LC/MS tuning mix (*m*/*z*, 1/K0:622.0289, 0.9848 Vs cm^–2^; 922.0097, 1.1895 Vs cm^–2^; and 1221.9906, 1.3820 Vs cm^–2^).

Data processing, protein identification, and relative quantification analyses were performed using the PEAKS studio software, version 10.6 (Bioinformatics Solutions Inc., Waterloo, ON). The processing parameters included cysteine carbamidomethyltion as a fixed amino acid modification, whereas oxidation of methionine and acetylation of the N-terminal region were considered as variable modifications. Trypsin was used as the proteolytic enzyme, with a maximum of two possible cleavage errors. The minimum size for peptides was seven amino acids. The ion mass deviation tolerance for peptides and fragments was set to 20 ppm and 0.05 Da, respectively.

A maximum FDR of 1% was used to identify peptides and proteins, considering at least one unique peptide for protein identification as the criterion. All proteins were identified with a confidence level of ≥95% using the PEAKS software algorithm and searching within the UniProt database for *E. coli* (taxon ID 562) and *Glycine max* (taxon ID 3847).

Proteomics results were filtered using the Perseus software. Proteins in the matrix identified only by one modification site, as well as those identified by the reverse database, and possible contaminants were excluded from subsequent analyses. Proteins were filtered so that only those with values >0 in at least 50% of the samples from at least one of the groups remained in the matrix. Subsequently, a script in the R programming language (https://www.R-project.org/) was used to refine the filter based on the percentage of protein presence in the groups and normalize the data by total ion count (TIC).

Furthermore, contaminant proteins present at levels higher than those of the target proteins, those detected using LC–MS, and exhibiting the highest area peak on the mass spectrum were analyzed. For this purpose, the proteins that presented the highest peak area were initially classified in descending order; thus, the first proteins are those that are the most abundant in the samples. The 10 most abundant proteins were selected from each sample, generating five tables with 10 proteins, each one representing the most abundant proteins in LegH A, C1, C2, and C3 and nsHb. These five tables revealed the proteins present in all or at least 3–4 samples. A unique table with the most abundant proteins observed in LegH A, C1, C2, and C3 and nsHb overall was then created. The detected proteins were filtered by those that presented the highest peak areas in descending order in each sample. A table was created with the contaminating proteins found most abundantly in all or 3–4 samples.

### 4.6 Assessment of LegH and Hb Peroxidase Activity

To verify whether LegH and nsHb presented pseudoperoxidase activity,^54^ 10 μL of purified and dialyzed LegH and nsHb at 400, 200, 100, and 50 μg/mL were added to 100 μL of 3,3′,5,5′-tetramethylbenzidine (TMB) liquid substrate system for ELISA (Cat no. T0440, Merck) in 96 clear flat-bottomed well plates. The absorbance was then read at 370 nm in a spectrophotometer at 25 °C every minute for 60 min to confirm the peroxidase activity based on the conversion of TMB into a blue product.

### 4.7 Heme Binding of LegHs and nsHb

Heme binding of LegHs and nsHb was assessed by adding 50 μL of purified and dialyzed proteins, water (blank), or calibrator at 200 μL of heme reagent (Cat no. MAK316, Merck) in 96 clear flat-bottomed well plates, and the absorbance was measured at 400 nm at 25 °C. Heme concentration was calculated using the equation given in the manufacturer’s instructions.

### 4.8 Pepsin Digestibility Assay

To assess the digestibility of LegHs and nsHb produced in the CFSs, these proteins were digested with pepsin (10 U/μg of LegH, Cat no. P7012, Merck) in an SGF containing 0.084 N HCl and 35 mM NaCl at pH 2.0 and 37 °C for 0, 1, 5, 25, and 50 min. After the defined durations, 20 μL of SGL containing each protein was recovered and mixed with 7 μL of 200 mM NaOHCO_3_ at pH 11 to inactivate pepsin and stop the digestion. These samples were denatured and subjected to SDS-PAGE as described previously, and the digestion of LegHs and nsHb was evaluated.

### 4.9 Statistical Analysis

Prism GraphPad was used to plot data, create graphs, and calculate statistical tests. Student’s *t*-test and two-tailed ANOVA were used to calculate the *p*-value for quantitative data between two groups and three or more groups, respectively.

## Supplementary Material



## Notes

Includes: vector constructs and alignment of soybean Hbs, a schematic for medium-scale cell-free protein synthesis, associated peroxidase activity data and mass spectrometry data of the synthesized proteins (PDF)
