# Supplementary material for: Cell-Free Production of Soybean Leghemoglobins and Nonsymbiotic Hemoglobin
Source: ACS Synth Biol. 2025 Aug 12;14(9):3445–56. doi: 10.1021/acssynbio.5c00197 (PMC12455655; doi:10.1021/acssynbio.5c00197)
Supplement: Supplementary file 1 [file sb5c00197_si_001.pdf]

Supporting Information

Cell-free production of soybean leghemoglobins and nonsymbiotic hemoglobin

Amanda P. Rocha[1], Mariele A. Palmeiras[1], Marco Antônio de Oliveira[1], Lilian H. Florentino[1], Thais R. Cataldi[2], Daniela M. de C. Bittencourt[1], Carlos A. Labate[2], Gracia M. S. Rosinha[1], Elibio L. Rech[1]*.

1 Embrapa Genetic Resources and Biotechnology / National Institute of Science and Technology - Synthetic Biology, Parque Estação Biologica, PqEB, Av. W5 Norte (final), Brasília, DF, 70770-917, Brazil, Norte (final), Brasília, DF, 70770-917, Brazil.

2 Max Feffer Laboratory of Plant Genetics - EMU, Department of Genetics, Luiz de Queiroz College of Agriculture, University of São Paulo, Av. Pádua Dias 11, Piracicaba – SP, 13418-900, Brazil.

*Corresponding author: elibio.rech@embrapa.br (orcid.org/0000-0001-8588-0673)

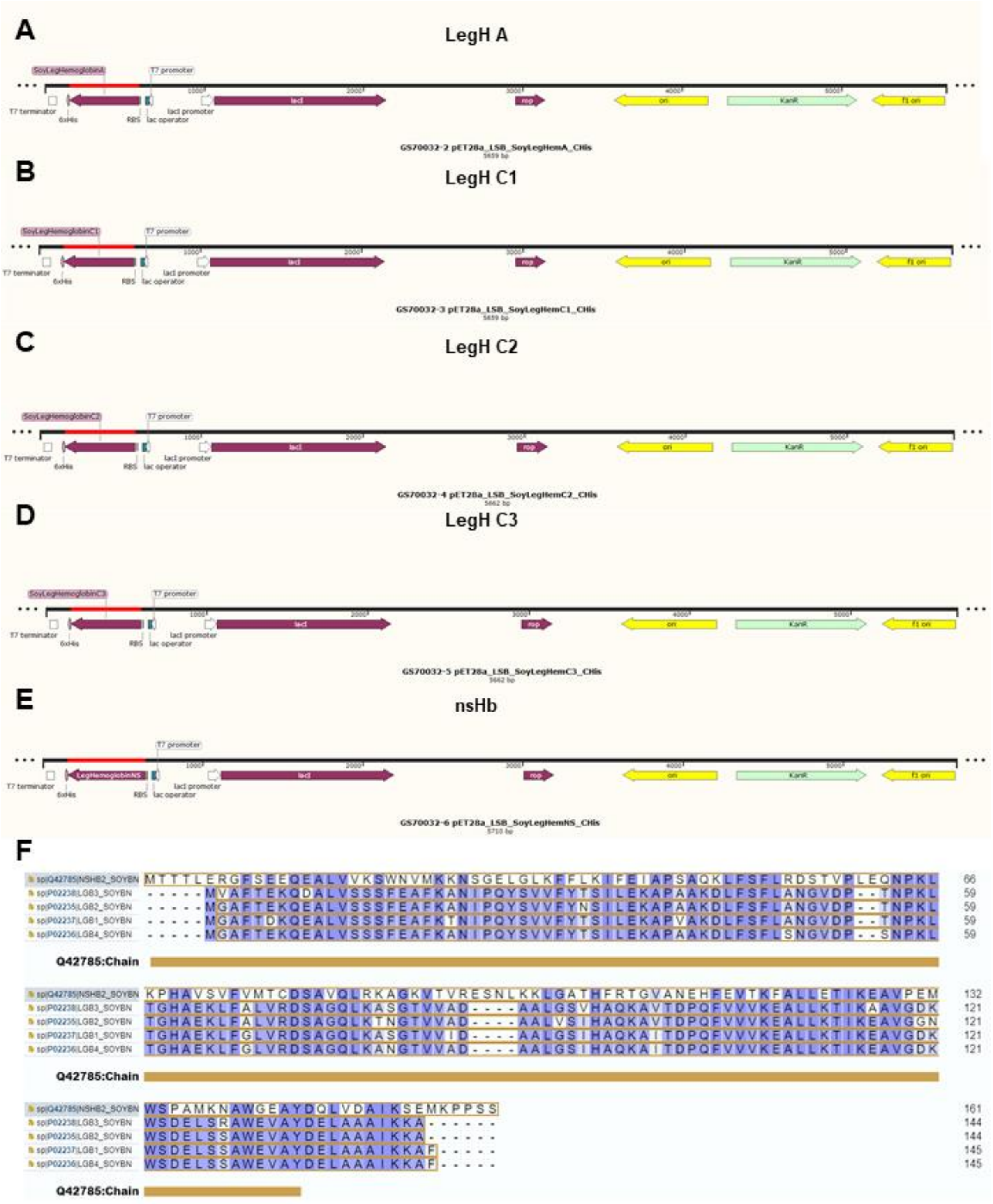

Figure S1. Vector constructions and alignment of soybean (Glycine max) Hbs. DNA sequences from soy LegH A (A), C1 (B), C2 (C), C3 (D) and nsHb (E) were cloned into the pET28a vector containing T7 promoter, ribosome binding site (RBS), T7 terminator and 6xHis-tag. Amino acids sequences from proteins LegH A, C1, C2, C3 and nsHb were aligned in Uniprot database showing conserved amino acids between different proteins (F).

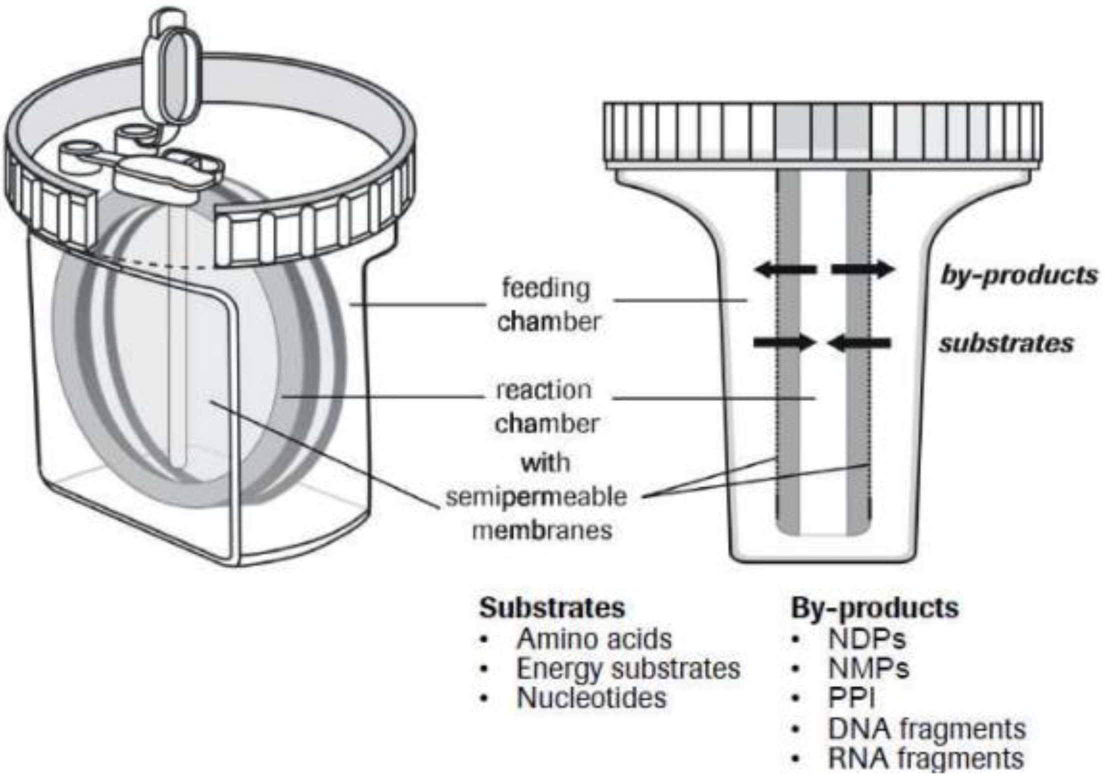

Figure S2. Schema of the RTS 500 Proteomaster device from Rabbit Biotechnology to produce medium-scale CFS using CECF system (cat #BR1400201). Illustration obtained from the manufacturer manual.

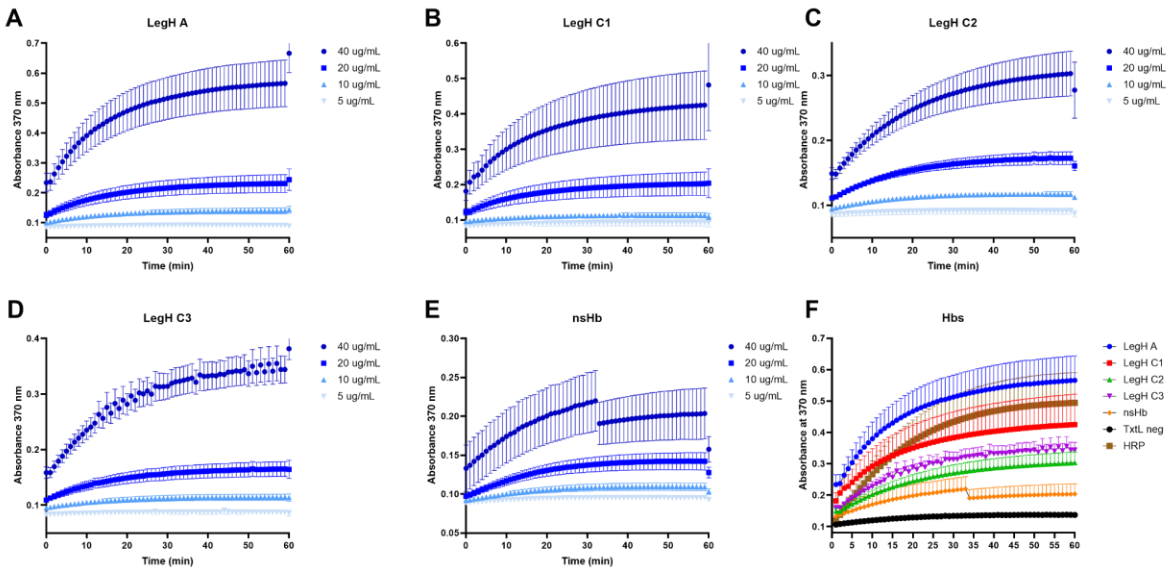

Figure S3: Soy Hbs from the cell-free system showed peroxidase activity. Hbs produced by the cell -free system were purified, dialyzed and then concentrated. These proteins were then mixed with 3,3',5,5'-Tetramethylbenzidine (TMB) liquid substrate and the absorbance was read at 370 nm every 1 minute for 1 hour to confirm the peroxidase activity through the conversion of TMB into a blue product. Peroxidase activity kinetics by the action of 40, 20, 10 and 5 µg/mL of LegH A (A), C1 (B), C2 (C), C3 (D) and Hb NS (E). Peroxidase activity kinetic with 40 µg/mL of LegH A, C1, C2, C3 and nsHb (F). Line graphs regarding means and standard error from four independent experiments performed in duplicate.

Table S1: Mass spectrometry data of the synthesized proteins.

Hb	Peptide	1E+07	Mass	Length	ppm	m/z	RT	1/k0 Range	Area A	Area C1	Area C2	Area C3	Area NS	Fraction	Precursor Id	Source File	#Feature	#Feature A	#Feature C1	#Feature C2	#Feature C3	#Feature NS	Accession	PTM	AScore	Found By
LegH_A	VAFTEKQDALVSSSFEAFK	67,24	21030522	19	29	7020293	91,76	0.9022-0.9308	0					1	52438	20240724_A_Slot1-2_1_4462.d	0	0	0	0	0	0	sp|P02238|LGB3_SOYBN:SoyLegHA|SoyLegHA|SoyLegHA			PEAKS DB
LegH_A	QDALVSSSFEAFK	114,8	14276932	13	17	7148578	82,76	0.9786-1.0069	1532600000					1	47795	20240724_A_Slot1-2_1_4462.d	3	3	0	0	0	0	sp|P02238|LGB3_SOYBN:SoyLegHA|SoyLegHA|SoyLegHA			PEAKS DB
LegH_A	ANIPQYSVVFYTSILEK	116,9	19710353	17	1	9865287	102,97	1.1797-1.2074	2476500		14854000			3	86855	20240724_C2_Slot1-4_1_4466.d	2	1	0	1	0	0	sp|P02238|LGB3_SOYBN:SoyLegHA|SoyLegHA|SoyLegHA:SoyLegHC2|SoyLegHC2|SoyLegHC2:sp|P02236|LGB4_SOYBN			PEAKS DB
LegH_A	DLFSFLANGVDPTNPK	171	17338624	16	24	8679434	104,45	1.0836-1.1116	25758000	147520000	3253700	256370000	6892600	4	70065	20240724_C3_Slot1-5_1_4468.d	8	1	3	1	2	1	SoyLegHC1|SoyLegHC1|SoyLegHC1:sp|P02235|LGB2_SOYBN:SoyLegHC3|SoyLegHC3|SoyLegHC3:C6T469|C6T469_SOYBN:sp|P02238|LGB3_SOYBN:SoyLegHA|SoyLegHA|SoyLegHA:sp|P02237|LGB1_SOYBN			PEAKS DB
LegH_A	LTGHAEKLFALVR	120,3	14538405	13	18	7279318	78,35	1.0903-1.1184	3388000	800070000				2	45461	20240724_C1_Slot1-3_1_4464.d	5	2	3	0	0	0	SoyLegHC1|SoyLegHC1|SoyLegHC1:sp|P02235|LGB2_SOYBN:sp|P02238|LGB3_SOYBN:SoyLegHA|SoyLegHA|SoyLegHA			PEAKS DB
LegH_A	LTGHAEKLFALVRDSAGQLK	69,25	21531956	20	6	7187425	100,63	0.9775-1.0058	74287000	332430000				2	64261	20240724_C1_Slot1-3_1_4464.d	5	1	4	0	0	0	SoyLegHC1|SoyLegHC1|SoyLegHC1:sp|P02235|LGB2_SOYBN:sp|P02238|LGB3_SOYBN:SoyLegHA|SoyLegHA|SoyLegHA			PEAKS DB
LegH_A	DSAGQLKASGTVVADAALGSVHAQK	145,1	23802346	25	20	7944233	78,24	1.0915-1.1195	36669000					1	45237	20240724_A_Slot1-2_1_4462.d	1	1	0	0	0	0	sp|P02238|LGB3_SOYBN:SoyLegHA|SoyLegHA|SoyLegHA			PEAKS DB
LegH_A	ASGTVVADAALGSVHAQK	170	16808794	18	-34	8414472	56,46	1.1697-1.1975	8001900000	388630000	282350000	39474000	11053000	1	30309	20240724_A_Slot1-2_1_4462.d	80	71	3	3	2	1	sp|P02238|LGB3_SOYBN:SoyLegHA|SoyLegHA|SoyLegHA			PEAKS DB
LegH_A	AVTDPQFVVVK	103,8	12016707	11	-22	6018438	68	0.8908-0.9194	801620000	3398300000	7641600			2	36970	20240724_C1_Slot1-3_1_4464.d	6	2	3	1	0	0	SoyLegHC1|SoyLegHC1|SoyLegHC1:sp|P02235|LGB2_SOYBN:sp|P02238|LGB3_SOYBN:SoyLegHA|SoyLegHA|SoyLegHA			PEAKS DB
LegH_A	AVTDPQFVVVKEALLK	127	17560134	16	19	8790193	95,87	1.1240-1.1519	20919000	467460000	766440			2	59925	20240724_C1_Slot1-3_1_4464.d	4	1	2	1	0	0	SoyLegHC1|SoyLegHC1|SoyLegHC1:sp|P02235|LGB2_SOYBN:sp|P02238|LGB3_SOYBN:SoyLegHA|SoyLegHA|SoyLegHA			PEAKS DB
LegH_A	AAVGDKWSDELSR	92,61	14326946	13	2	7173574	50,96	0.9570-0.9854	175350000					1	26029	20240724_A_Slot1-2_1_4462.d	1	1	0	0	0	0	sp|P02238|LGB3_SOYBN:SoyLegHA|SoyLegHA|SoyLegHA			PEAKS DB
LegH_A	AWEVAYDELAAAIK	106,6	15487823	14	60	7754059	100,63	1.0645-1.0926	1031700					1	56644	20240724_A_Slot1-2_1_4462.d	1	1	0	0	0	0	sp|P02238|LGB3_SOYBN:SoyLegHA|SoyLegHA|SoyLegHA			PEAKS DB
LegH_A	AWEVAYDELAAAIKK	126,8	16768773	15	16	8394504	97,93	1.1060-1.1340	7694600					1	55042	20240724_A_Slot1-2_1_4462.d	1	1	0	0	0	0	sp|P02238|LGB3_SOYBN:SoyLegHA|SoyLegHA|SoyLegHA			PEAKS DB
LegH_C1	MGAFTEK	49,21	7823633	7	-51	3921885	21,8	0.7139-0.7430		0				2	6132	20240724_C1_Slot1-3_1_4464.d	0	0	0	0	0	0	SoyLegHC1|SoyLegHC1|SoyLegHC1:sp|P02235|LGB2_SOYBN:SoyLegHC2|SoyLegHC2|SoyLegHC2:sp|P02236|LGB4_SOYBN			PEAKS DB
LegH_C1	M(+15.99)GAFTEKQEALVSSSFEAFK	104	22220564	20	70	7417009	91,41	0.9422-0.9707		5132500				2	57219	20240724_C1_Slot1-3_1_4464.d	1	0	1	0	0	0	SoyLegHC1|SoyLegHC1|SoyLegHC1:sp|P02235|LGB2_SOYBN:SoyLegHC2|SoyLegHC2|SoyLegHC2:sp|P02236|LGB4_SOYBN	Oxidation (M)	M1:Oxidation (M):1000.00	PEAKS DB
LegH_C1	GAFTEKQEALVSSSFEAFK	97,28	20750210	19	-7	6926833	91,6	0.8575-0.8862		974190000	1360100000			2	57368	20240724_C1_Slot1-3_1_4464.d	10	0	4	6	0	0	SoyLegHC1|SoyLegHC1|SoyLegHC1:sp|P02235|LGB2_SOYBN:SoyLegHC2|SoyLegHC2|SoyLegHC2:sp|P02236|LGB4_SOYBN			PEAKS DB
LegH_C1	QEALVSSSFEAFK	132,5	14417089	13	5	7218647	81,77	0.9741-1.0024		3201400000	1179800000	443190000	3573100	3	62809	20240724_C2_Slot1-4_1_4466.d	21	0	10	8	2	1	SoyLegHC1|SoyLegHC1|SoyLegHC1:sp|P02235|LGB2_SOYBN:SoyLegHC3|SoyLegHC3|SoyLegHC3:C6T469|C6T469_SOYBN:SoyLegHC2|SoyLegHC2|SoyLegHC2:sp|P02236|LGB4_SOYBN:sp|P02237|LGB1_SOYBN			PEAKS DB
LegH_C1	ANIPQYSVVFYNSILEKAPAAK	55,87	24222896	22	-37	8084374	99,95	0.9422-0.9707		914660				2	63893	20240724_C1_Slot1-3_1_4464.d	1	0	1	0	0	0	SoyLegHC1|SoyLegHC1|SoyLegHC1:sp|P02235|LGB2_SOYBN			PEAKS DB
LegH_C1	ANIPQYSVVFYNSILEK	129	19840305	17	-5	9930261	101,29	1.1563-1.1841		69156000				2	64790	20240724_C1_Slot1-3_1_4464.d	1	0	1	0	0	0	SoyLegHC1|SoyLegHC1|SoyLegHC1:sp|P02235|LGB2_SOYBN			PEAKS DB
LegH_C1	DLFSFLANGVDPTNPK	171	17338624	16	24	8679434	104,45	1.0836-1.1116	25758000	147520000	3253700	256370000	6892600	4	70065	20240724_C3_Slot1-5_1_4468.d	8	1	3	1	2	1	SoyLegHC1|SoyLegHC1|SoyLegHC1:sp|P02235|LGB2_SOYBN:SoyLegHC3|SoyLegHC3|SoyLegHC3:C6T469|C6T469_SOYBN:sp|P02238|LGB3_SOYBN:SoyLegHA|SoyLegHA|SoyLegHA:sp|P02237|LGB1_SOYBN			PEAKS DB
LegH_C1	LTGHAEKLFALVR	120,3	14538405	13	18	7279318	78,35	1.0903-1.1184	3388000	800070000				2	45461	20240724_C1_Slot1-3_1_4464.d	5	2	3	0	0	0	SoyLegHC1|SoyLegHC1|SoyLegHC1:sp|P02235|LGB2_SOYBN:sp|P02238|LGB3_SOYBN:SoyLegHA|SoyLegHA|SoyLegHA			PEAKS DB
LegH_C1	LTGHAEKLFALVRDSAGQLK	69,25	21531956	20	6	7187425	100,63	0.9775-1.0058	74287000	332430000				2	64261	20240724_C1_Slot1-3_1_4464.d	5	1	4	0	0	0	SoyLegHC1|SoyLegHC1|SoyLegHC1:sp|P02235|LGB2_SOYBN:sp|P02238|LGB3_SOYBN:SoyLegHA|SoyLegHA|SoyLegHA			PEAKS DB
LegH_C1	LTGHAEKLFALVRDSAGQLKTNGTVVADAALVSIHA	66,67	39291487	38	92	7868475	105,24	1.0408-1.0690		0	0			2	67837	20240724_C1_Slot1-3_1_4464.d	0	0	0	0	0	0	SoyLegHC1|SoyLegHC1|SoyLegHC1:sp|P02235|LGB2_SOYBN			PEAKS DB
LegH_C1	LFALVRDSAGQLK	88,95	14168088	13	31	7094168	71,72	1.0239-1.0521		11613000				2	40546	20240724_C1_Slot1-3_1_4464.d	2	0	2	0	0	0	SoyLegHC1|SoyLegHC1|SoyLegHC1:sp|P02235|LGB2_SOYBN:sp|P02238|LGB3_SOYBN:SoyLegHA|SoyLegHA|SoyLegHA			PEAKS DB
LegH_C1	DSAGQLK	47,88	7173657	7	55	3596936	4,22	0.6952-0.7244		0				2	447	20240724_C1_Slot1-3_1_4464.d	0	0	0	0	0	0	SoyLegHC1|SoyLegHC1|SoyLegHC1:sp|P02235|LGB2_SOYBN:SoyLegHC3|SoyLegHC3|SoyLegHC3:C6T469|C6T469_SOYBN:sp|P02238|LGB3_SOYBN:SoyLegHA|SoyLegHA|SoyLegHA:SoyLegHC2|SoyLegHC2|SoyLegHC2:sp|P02236|LGB4_SOYBN:sp|P02237|LGB1_SOYBN			PEAKS DB
LegH_C1	AVTDPQFVVVK	103,8	12016707	11	-22	6018438	68	0.8908-0.9194	801620000	3398300000	7641600			2	36970	20240724_C1_Slot1-3_1_4464.d	6	2	3	1	0	0	SoyLegHC1|SoyLegHC1|SoyLegHC1:sp|P02235|LGB2_SOYBN:sp|P02238|LGB3_SOYBN:SoyLegHA|SoyLegHA|SoyLegHA			PEAKS DB
LegH_C1	AVTDPQFVVVKEALLK	127	17560134	16	19	8790193	95,87	1.1240-1.1519	20919000	467460000	766440			2	59925	20240724_C1_Slot1-3_1_4464.d	4	1	2	1	0	0	SoyLegHC1|SoyLegHC1|SoyLegHC1:sp|P02235|LGB2_SOYBN:sp|P02238|LGB3_SOYBN:SoyLegHA|SoyLegHA|SoyLegHA			PEAKS DB
LegH_C1	TIKEAVGGNWSDELSSAWEVAYDELAAAIKK	49,03	33506670	31	16	11179026	105,3	1.2473-1.2748		0				2	67874	20240724_C1_Slot1-3_1_4464.d	0	0	0	0	0	0	SoyLegHC1|SoyLegHC1|SoyLegHC1:sp|P02235|LGB2_SOYBN			PEAKS DB
LegH_C1	TIKEAVGGNWSDELSSAWEVAYDELAAAIK	102,2	32225720	30	103	10752134	105,63	1.0690-1.0971		0				2	68046	20240724_C1_Slot1-3_1_4464.d	0	0	0	0	0	0	SoyLegHC1|SoyLegHC1|SoyLegHC1:sp|P02235|LGB2_SOYBN			PEAKS DB
LegH_C1	EAVGGNWSDELSSAWEVAYDELAAAIKK	112,2	30084402	28	53	10038301	105,61	1.1730-1.2008		8062300				2	68020	20240724_C1_Slot1-3_1_4464.d	1	0	1	0	0	0	SoyLegHC1|SoyLegHC1|SoyLegHC1:sp|P02235|LGB2_SOYBN			PEAKS DB
LegH_C1	EAVGGNWSDELSSAWEVAYDELAAAIK	63,41	28803452	27	-29	14411816	106,07	1.3603-1.3875		0				2	68196	20240724_C1_Slot1-3_1_4464.d	0	0	0	0	0	0	SoyLegHC1|SoyLegHC1|SoyLegHC1:sp|P02235|LGB2_SOYBN			PEAKS DB
LegH_C2	GAFTEKQEALVSSSFEAFK	97,28	20750210	19	-7	6926833	91,6	0.8575-0.8862		974190000	1360100000			2	57368	20240724_C1_Slot1-3_1_4464.d	10	0	4	6	0	0	SoyLegHC1|SoyLegHC1|SoyLegHC1:sp|P02235|LGB2_SOYBN:SoyLegHC2|SoyLegHC2|SoyLegHC2:sp|P02236|LGB4_SOYBN			PEAKS DB
LegH_C2	QEALVSSSFEAFK	132,5	14417089	13	5	7218647	81,77	0.9741-1.0024		3201400000	1179800000	443190000	3573100	3	62809	20240724_C2_Slot1-4_1_4466.d	21	0	10	8	2	1	SoyLegHC1|SoyLegHC1|SoyLegHC1:sp|P02235|LGB2_SOYBN:SoyLegHC3|SoyLegHC3|SoyLegHC3:C6T469|C6T469_SOYBN:SoyLegHC2|SoyLegHC2|SoyLegHC2:sp|P02236|LGB4_SOYBN:sp|P02237|LGB1_SOYBN			PEAKS DB
LegH_C2	ANIPQYSVVFYTSILEK	116,9	19710353	17	1	9865287	102,97	1.1797-1.2074	2476500		14854000			3	86855	20240724_C2_Slot1-4_1_4466.d	2	1	0	1	0	0	sp|P02238|LGB3_SOYBN:SoyLegHA|SoyLegHA|SoyLegHA:SoyLegHC2|SoyLegHC2|SoyLegHC2:sp|P02236|LGB4_SOYBN			PEAKS DB
LegH_C2	DLFSFLSNGVDPSNPK	144,8	17358417	16	19	8689330	103,29	1.0656-1.0937			85048000			3	87209	20240724_C2_Slot1-4_1_4466.d	1	0	0	1	0	0	SoyLegHC2|SoyLegHC2|SoyLegHC2:sp|P02236|LGB4_SOYBN			PEAKS DB
LegH_C2	LTGHAEKLFGLVR	109,8	14398248	13	11	7209229	73,72	1.0375-1.0656			338480000	173060000		4	44845	20240724_C3_Slot1-5_1_4468.d	6	0	0	3	3	0	SoyLegHC3|SoyLegHC3|SoyLegHC3:C6T469|C6T469_SOYBN:SoyLegHC2|SoyLegHC2|SoyLegHC2:sp|P02236|LGB4_SOYBN:sp|P02237|LGB1_SOYBN			PEAKS DB
LegH_C2	LTGHAEKLFGLVRDSAGQLKANGTVVADAALGSIHA	89,39	38430754	38	14	7696263	104,56	1.0307-1.0589			128060000	0		3	88589	20240724_C2_Slot1-4_1_4466.d	1	0	0	1	0	0	SoyLegHC2|SoyLegHC2|SoyLegHC2:sp|P02236|LGB4_SOYBN			PEAKS DB
LegH_C2	DSAGQLKANGTVVADAALGSIHAQK	123,9	24212612	25	22	8080991	80,77	1.0903-1.1184			463480000			3	62089	20240724_C2_Slot1-4_1_4466.d	4	0	0	4	0	0	SoyLegHC2|SoyLegHC2|SoyLegHC2:sp|P02236|LGB4_SOYBN			PEAKS DB
LegH_C2	ANGTVVADAALGSIHAQK	166,7	17219060	18	-16	8619621	64,11	1.2008-1.2285			17968000000	48434000	8757100	3	44671	20240724_C2_Slot1-4_1_4466.d	37	0	0	34	2	1	SoyLegHC2|SoyLegHC2|SoyLegHC2:sp|P02236|LGB4_SOYBN			PEAKS DB
LegH_C2	AITDPQFVVVK	95,65	12156863	11	-15	6088515	74,92	0.8977-0.9262		37133000	314460000	713610000		4	45754	20240724_C3_Slot1-5_1_4468.d	4	0	1	1	2	0	SoyLegHC3|SoyLegHC3|SoyLegHC3:C6T469|C6T469_SOYBN:SoyLegHC2|SoyLegHC2|SoyLegHC2:sp|P02236|LGB4_SOYBN:sp|P02237|LGB1_SOYBN			PEAKS DB
LegH_C2	AITDPQFVVVKEALLK	119,9	17700291	16	-2	8860245	97,6	1.1318-1.1597			27633000	14752000		4	63581	20240724_C3_Slot1-5_1_4468.d	2	0	0	1	1	0	SoyLegHC3|SoyLegHC3|SoyLegHC3:C6T469|C6T469_SOYBN:SoyLegHC2|SoyLegHC2|SoyLegHC2:sp|P02236|LGB4_SOYBN:sp|P02237|LGB1_SOYBN			PEAKS DB
LegH_C3	M(+15.99)GAFTDKQEALVSSSFEAFK	99,55	22080408	20	-37	7370205	91,34	0.8942-0.9228				0		4	58856	20240724_C3_Slot1-5_1_4468.d	0	0	0	0	0	0	SoyLegHC3|SoyLegHC3|SoyLegHC3:C6T469|C6T469_SOYBN:sp|P02237|LGB1_SOYBN	Oxidation (M)	M1:Oxidation (M):1000.00	PEAKS DB
LegH_C3	MGAFTDKQEALVSSSFEAFK	82,73	21920459	20	-42	7316885	95,97	0.8942-0.9228				0		4	62110	20240724_C3_Slot1-5_1_4468.d	0	0	0	0	0	0	SoyLegHC3|SoyLegHC3|SoyLegHC3:C6T469|C6T469_SOYBN:sp|P02237|LGB1_SOYBN			PEAKS DB
LegH_C3	GAFTDKQEALVSSSFEAFK	118,3	20610054	19	-18	6880101	92,03	0.8598-0.8885			0	260200000		4	59020	20240724_C3_Slot1-5_1_4468.d	5	0	0	0	5	0	SoyLegHC3|SoyLegHC3|SoyLegHC3:C6T469|C6T469_SOYBN:sp|P02237|LGB1_SOYBN			PEAKS DB
LegH_C3	TNIPQYSVVFYTSILEK	120,1	20010459	17	-16	10015319	103,07	1.1764-1.2041				52099000		4	68615	20240724_C3_Slot1-5_1_4468.d	1	0	0	0	1	0	SoyLegHC3|SoyLegHC3|SoyLegHC3:C6T469|C6T469_SOYBN:sp|P02237|LGB1_SOYBN			PEAKS DB
LegH_C3	TNIPQYSVVFYTSILEKAPVAK	95,78	24673362	22	11	12346808	102,62	1.4287-1.4558				0		4	68332	20240724_C3_Slot1-5_1_4468.d	0	0	0	0	0	0	SoyLegHC3|SoyLegHC3|SoyLegHC3:C6T469|C6T469_SOYBN:sp|P02237|LGB1_SOYBN			PEAKS DB
LegH_C3	DLFSFLANGVDPTNPK	171	17338624	16	24	8679434	104,45	1.0836-1.1116	25758000	147520000	3253700	256370000	6892600	4	70065	20240724_C3_Slot1-5_1_4468.d	8	1	3	1	2	1	SoyLegHC1|SoyLegHC1|SoyLegHC1:sp|P02235|LGB2_SOYBN:SoyLegHC3|SoyLegHC3|SoyLegHC3:C6T469|C6T469_SOYBN:sp|P02238|LGB3_SOYBN:SoyLegHA|SoyLegHA|SoyLegHA:sp|P02237|LGB1_SOYBN			PEAKS DB
LegH_C3	LTGHAEKLFGLVR	109,8	14398248	13	11	7209229	73,72	1.0375-1.0656			338480000	173060000		4	44845	20240724_C3_Slot1-5_1_4468.d	6	0	0	3	3	0	SoyLegHC3|SoyLegHC3|SoyLegHC3:C6T469|C6T469_SOYBN:SoyLegHC2|SoyLegHC2|SoyLegHC2:sp|P02236|LGB4_SOYBN:sp|P02237|LGB1_SOYBN			PEAKS DB
LegH_C3	LFGLVRDSAGQLK	63,26	14027932	13	44	7024092	72,3	1.0183-1.0465				2537600		4	43798	20240724_C3_Slot1-5_1_4468.d	1	0	0	0	1	0	SoyLegHC3|SoyLegHC3|SoyLegHC3:C6T469|C6T469_SOYBN:SoyLegHC2|SoyLegHC2|SoyLegHC2:sp|P02236|LGB4_SOYBN:sp|P02237|LGB1_SOYBN			PEAKS DB
LegH_C3	DSAGQLK	47,88	7173657	7	55	3596936	4,22	0.6952-0.7244		0				2	447	20240724_C1_Slot1-3_1_4464.d	0	0	0	0	0	0	SoyLegHC1|SoyLegHC1|SoyLegHC1:sp|P02235|LGB2_SOYBN:SoyLegHC3|SoyLegHC3|SoyLegHC3:C6T469|C6T469_SOYBN:sp|P02238|LGB3_SOYBN:SoyLegHA|SoyLegHA|SoyLegHA:SoyLegHC2|SoyLegHC2|SoyLegHC2:sp|P02236|LGB4_SOYBN:sp|P02237|LGB1_SOYBN			PEAKS DB
LegH_C3	DSAGQLKASGTVVIDAALGSIHGQK	128,2	24222815	25	42	8084404	97,07	1.0859-1.1139				25523000		4	63133	20240724_C3_Slot1-5_1_4468.d	1	0	0	0	1	0	SoyLegHC3|SoyLegHC3|SoyLegHC3:C6T469|C6T469_SOYBN			PEAKS DB
LegH_C3	ASGTVVIDAALGSIHGQK	200	17229264	18	-10	8624724	82,99	1.1841-1.2119	0		94733000	9921300000	103720000	4	52167	20240724_C3_Slot1-5_1_4468.d	39	0	0	1	37	1	SoyLegHC3|SoyLegHC3|SoyLegHC3:C6T469|C6T469_SOYBN			PEAKS DB
LegH_C3	AITDPQFVVVK	95,65	12156863	11	-15	6088515	74,92	0.8977-0.9262		37133000	314460000	713610000		4	45754	20240724_C3_Slot1-5_1_4468.d	4	0	1	1	2	0	SoyLegHC3|SoyLegHC3|SoyLegHC3:C6T469|C6T469_SOYBN:SoyLegHC2|SoyLegHC2|SoyLegHC2:sp|P02236|LGB4_SOYBN:sp|P02237|LGB1_SOYBN			PEAKS DB
LegH_C3	AITDPQFVVVKEALLK	119,9	17700291	16	-2	8860245	97,6	1.1318-1.1597			27633000	14752000		4	63581	20240724_C3_Slot1-5_1_4468.d	2	0	0	1	1	0	SoyLegHC3|SoyLegHC3|SoyLegHC3:C6T469|C6T469_SOYBN:SoyLegHC2|SoyLegHC2|SoyLegHC2:sp|P02236|LGB4_SOYBN:sp|P02237|LGB1_SOYBN			PEAKS DB
nsHB	GFSEEQEALVVK	148,7	13346718	12	7	6683464	61,97	0.9513-0.9797					2598300000	5	49467	20240724_NS_Slot1-6_1_4470.d	3	0	0	0	0	3	sp|Q42785|NSHB2_SOYBN:SoyLegHNS|SoyLegHNS|SoyLegHNS			PEAKS DB
nsHB	SWNVM(+15.99)KK	70,17	9074586	7	-45	4547345	37,18	0.7771-0.8056					51915000	3	3622	20240507_NS_Slot1-5_1_4327.d	3	0	0	0	0	3	|Leghemoglobin	Oxidation (M)	M5:Oxidation (M):1000.00	PEAKS DB
nsHB	DSTVPLEQNPK	51,09	12266143	11	59	6143206	32,04	0.9091-0.9376					50249000	5	18628	20240724_NS_Slot1-6_1_4470.d	2	0	0	0	0	2	sp|Q42785|NSHB2_SOYBN:SoyLegHNS|SoyLegHNS|SoyLegHNS			PEAKS DB
nsHB	KLGATHFR	64,53	9285242	8	-19	4652704	15,11	0.7940-0.8229					1317900000	5	4502	20240724_NS_Slot1-6_1_4470.d	2	0	0	0	0	2	sp|Q42785|NSHB2_SOYBN:SoyLegHNS|SoyLegHNS|SoyLegHNS			PEAKS DB
nsHB	LGATHFR	68,26	8004293	7	45	4012238	34,89	0.7152-0.7439					563570000	3	3266	20240507_NS_Slot1-5_1_4327.d	2	0	0	0	0	2	|Leghemoglobin			PEAKS DB
nsHB	TGVANEHFEVTK	122	13306517	12	15	6663369	35,95	0.9399-0.9684					721090000	5	22191	20240724_NS_Slot1-6_1_4470.d	3	0	0	0	0	3	sp|Q42785|NSHB2_SOYBN:SoyLegHNS|SoyLegHNS|SoyLegHNS			PEAKS DB
nsHB	NAWGEAYDQLVDAIK	125,7	16918154	15	-26	8469163	101,69	1.0656-1.0937					36654000	5	94931	20240724_NS_Slot1-6_1_4470.d	1	0	0	0	0	1	sp|Q42785|NSHB2_SOYBN:SoyLegHNS|SoyLegHNS|SoyLegHNS			PEAKS DB
